# Poly[[penta­aqua­(μ_4_-pyridine-2,4,6-tri­carboxyl­ato)(μ_3_-pyridine-2,4,6-tri­carboxyl­ato)diterbium(III)] mono­hydrate]

**DOI:** 10.1107/S1600536812032898

**Published:** 2012-07-28

**Authors:** Xiao-Ke Yu, Hong-Lin Zhu

**Affiliations:** a Center of Applied Solid State Chemistry Research, Ningbo University, Ningbo, Zhejiang 315211, People’s Republic of China

## Abstract

The three-dimensional title coordination polymer, {[Tb_2_(C_8_H_2_NO_6_)_2_(H_2_O)_5_]·H_2_O}_*n*_, was hydro­thermally synthesized by reacting the corresponding rare-earth salt with pyridine-2,4,6-tricarb­oxy­lic acid (H_3_ptc). There are two independent Tb^III^ atoms in the structure, one of which is nine-coordinated, forming a monocapped NO_8_ square-anti­prism and the other is eight-coordinated exhibiting a 4,4-bicapped NO_7_ trigonal–prismatic environment. The complex units are inter­connected through the ptc^3−^ anions acting in different coordination modes, resulting in a three-dimensional coordin­ation polymer. The crystal structure features extensive O—H⋯O hydrogen bonds.

## Related literature

For general background to the design and synthesis of metal organic frameworks (MOFs) with lanthanides, see: Wang *et al.* (2007 ▶); Fu & Xu (2008 ▶); Das *et al.* (2009 ▶). For related structures, see: Lin *et al.* (2011 ▶). 
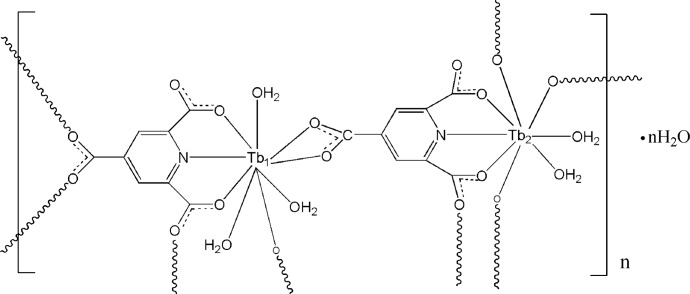


## Experimental

### 

#### Crystal data

[Tb_2_(C_8_H_2_NO_6_)_2_(H_2_O)_5_]·H_2_O
*M* *_r_* = 842.15
Monoclinic, 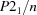

*a* = 18.426 (4) Å
*b* = 6.9082 (14) Å
*c* = 18.583 (4) Å
β = 111.98 (3)°
*V* = 2193.6 (8) Å^3^
*Z* = 4
Mo *K*α radiation
μ = 6.50 mm^−1^
*T* = 293 K
0.38 × 0.34 × 0.31 mm

#### Data collection

Rigaku R-AXIS RAPID diffractometer
Absorption correction: multi-scan (*ABSCOR*; Higashi, 1995 ▶) *T*_min_ = 0.101, *T*_max_ = 0.128
20352 measured reflections
4912 independent reflections
4764 reflections with *I* > 2σ(*I*)
*R*_int_ = 0.041

#### Refinement

*R*[*F* ^2^ > 2σ(*F* ^2^)] = 0.020
*wR*(*F* ^2^) = 0.045
*S* = 1.20
4912 reflections
344 parameters
H-atom parameters constrained
Δρ_max_ = 1.07 e Å^−3^
Δρ_min_ = −1.34 e Å^−3^

### 

Data collection: *RAPID-AUTO* (Rigaku, 1998 ▶); cell refinement: *RAPID-AUTO*; data reduction: *CrystalStructure* (Rigaku/MSC, 2004 ▶); program(s) used to solve structure: *SHELXS97* (Sheldrick, 2008 ▶); program(s) used to refine structure: *SHELXL97* (Sheldrick, 2008 ▶); molecular graphics: *SHELXTL* (Sheldrick, 2008 ▶); software used to prepare material for publication: *SHELXL97*.

## Supplementary Material

Crystal structure: contains datablock(s) global, I. DOI: 10.1107/S1600536812032898/bg2463sup1.cif

Structure factors: contains datablock(s) I. DOI: 10.1107/S1600536812032898/bg2463Isup2.hkl

Additional supplementary materials:  crystallographic information; 3D view; checkCIF report

## Figures and Tables

**Table 1:** Selected bond lengths (Å)

Tb1—O1^i^	2.508 (2)
Tb1—O2	2.400 (2)
Tb1—O6	2.378 (2)
Tb1—O7	2.426 (2)
Tb1—O8	2.406 (2)
Tb1—O9	2.431 (2)
Tb1—O12	2.590 (2)
Tb1—O13	2.489 (2)
Tb1—N1	2.534 (2)
Tb2—O3^ii^	2.365 (2)
Tb2—O4^iii^	2.387 (2)
Tb2—O10^iv^	2.336 (2)
Tb2—O11	2.403 (2)
Tb2—O15	2.384 (2)
Tb2—O16	2.430 (2)
Tb2—O17	2.358 (2)
Tb2—N2	2.507 (2)

Symmetry codes: (i) 
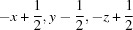
; (ii) 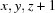; (iii) 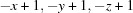; (iv) 
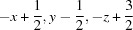
.

**Table 2:** Hydrogen-bond geometry (Å, °)

*D*—H⋯*A*	*D*—H	H⋯*A*	*D*⋯*A*	*D*—H⋯*A*
O7—H7*A*⋯O14^v^	0.84	1.86	2.700 (3)	174.4
O7—H7*B*⋯O2^i^	0.87	1.80	2.651 (3)	164.8
O8—H8*A*⋯O12^iii^	0.85	1.89	2.741 (3)	176.0
O8—H8*B*⋯O9^vi^	0.86	2.39	2.844 (3)	113.0
O9—H9*A*⋯O5^v^	0.85	2.56	3.057 (3)	119.0
O9—H9*A*⋯O6^v^	0.85	1.86	2.711 (3)	176.0
O9—H9*B*⋯O5^v^	0.85	2.56	3.057 (3)	118.0
O16—H16*A*⋯O4^v^	0.85	2.30	3.101 (3)	156.2
O16—H16*B*⋯O15^vii^	0.81	1.96	2.766 (3)	171.0
O17—H17*A*⋯O18^iv^	0.87	1.84	2.705 (4)	173.9
O17—H17*B*⋯O5^viii^	0.82	1.95	2.759 (3)	163.6
O18—H18*A*⋯O14^ix^	0.87	2.04	2.911 (4)	175.7
O18—H18*B*⋯O13	0.83	1.92	2.745 (4)	167.1

Symmetry codes: (i) 
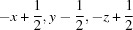
; (iii) 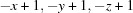; (iv) 
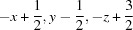
; (v) 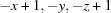; (vi) 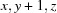; (vii) 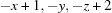; (viii) 
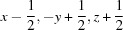
; (ix) 
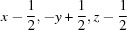
.

**Fig. 1.**
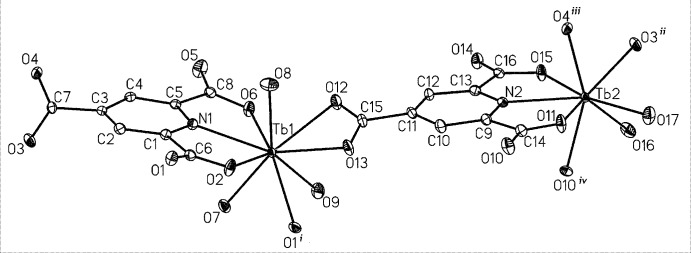
ORTEP view of the title compound, The dispalcement ellipsoids are drawn at 45% probability dispalcement ellipsoids. Symmetry codes: (i) -x + 1/2, y - 1/2, -z + 1/2; (ii) x, y, z + 1; (iii) -x + 1, -y + 1, -z + 1; (iv) -x + 1/2, y - 1/2, -z + 3/2.

**Fig. 2.**
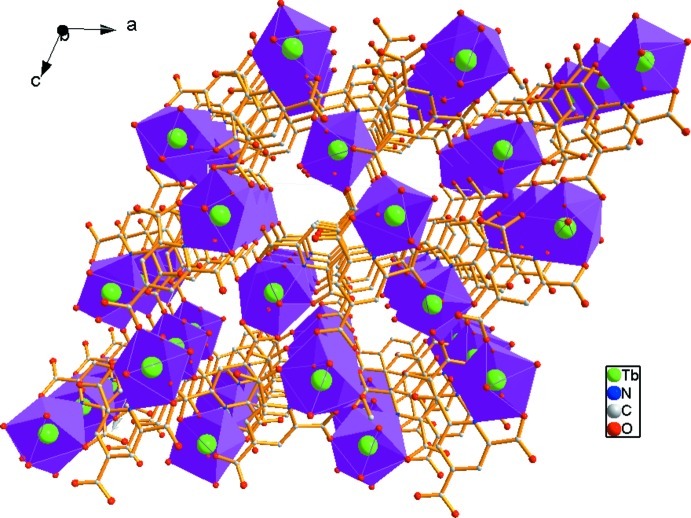
the three-dimensional structure of title complex.

## supplementary crystallographic information

### Comment

In recent years, the design and synthesis of metal organic frameworks (MOFs) with lanthanide have become an fascinating field due to their potential applications in luminescent materials, magnetic, catalyst and gas absorption (Wang *et al.*, 2007). Multicarboxylic acids were widely used as organic linkers in the syntheses of MOFs such as phthalic acid, trimesic acid and pyromellitic acid. In addition, pyridine-2,4,6-tricarboxylato(H_3_ptc) is a good building unit for constructing MFOs due to the existence of both N and O atoms in the ligands (Fu *et al.*, 2008). Another reason for choosing H_3_ptc is the inherent negative charge associated with them that helps in the charge compensation of the metal ion in the framework (Das *et al.*, 2009). At the same time, Lanthanide complexes with aromatic carboxylic acids show higher thermal or luminescence stabilities for practical application than other lanthanide complex systems. Thus we design and synthesis the title compound prepared from Tb_4_O_7_ and pyridine-2, 4, 6-tricarboxylic acid.

The asymmetric unit of [Tb_2_(H_2_O)_5_(ptc)_2_]_n_.nH_2_O consists of two Tb^3+^ ions (Tb1,Tb2), two ptc^3-^ ions, five aqua ligands, and a lattice water as illustrated in Fig. 1. Its worth to mention,The Tb1 atoms is nine-coordinated fashion by three aqua ligands (O7, O8 and O9) as well as three ptc ligands to generate a distorted monocapped squarean-tiprismatic DyNO8 chromophore with d(Tb1—N1) = 2.517 (2) Å and d(Tb1—O) = 2.366–2.566 Å, and Tb2 is eight-coordinated fashion by two aqua ligands (O4 and O9) as well as three ptc ligands texhibit a 4,4-bicapped trigonal prismatic TbNO7 chromophore with d(Tb2—N2) = 2.486 (2) Å and d(Tb2—O) = 2.320–2.415 Å, respectively (Lin *et al.*, 2011). Interestingly, the C7—O14 distance in pyridine-2, 4, 6-tricarboxylic acid is significantly shorter than that of corresponding C—O distance due to the the adjacent molecular's close packing. The three-dimensional polymer is constructed by the Tb_2_ building units through ptc^3-^ anions in different coordination modes.

### Experimental

Pale green powder of TbCl_3_.nH_2_O was obtained by slow evaporation of a solution of Tb_4_O_7_(0.25 mmol, 0.185 g) dissolved in 10 ml HCl(1 *M*) under water boiling condition. The freshly prepared TbCl_3_.nH_2_O, H_3_ptc(0.054 g, 0.25 mmol), malonic acid(0.026 g, 0.25 mmol),15 ml H_2_O and 1 ml NaOH(1 *M*) were sealed in a 23 ml Teflon-lined stainless autoclave, which was heated at 453 K for three days and thereafter cooled slowly to room temperature, and Pale green crystals were seperated by filtering and washing.

### Refinement

H atoms bonded to C atoms were palced in geometrically calculated position and were refined using a riding model, with *U*_iso_(H) = 1.2 *U*_eq_(C). H atoms attached to O atoms were found in a difference Fourier synthesis and were refined using a riding model, with the O—H distances fixed as initially found and with *U*_iso_(H) values set at 1.2 *U*eq(O).

### Crystal data

**Table:**

[Tb_2_(C_8_H_2_NO_6_)_2_(H_2_O)_5_]·H_2_O	*Z* = 4
*M**_r_* = 842.15	*F*(000) = 1600
Monoclinic, *P*2_1_/*n*	*D*_x_ = 2.550 Mg m^−^^3^
Hall symbol: -P 2yn	Mo *K*α radiation, λ = 0.71073 Å
*a* = 18.426 (4) Å	θ = 3.2–27.5°
*b* = 6.9082 (14) Å	µ = 6.50 mm^−^^1^
*c* = 18.583 (4) Å	*T* = 293 K
β = 111.98 (3)°	Block, colorless
*V* = 2193.6 (8) Å^3^	0.38 × 0.34 × 0.31 mm

### Data collection

**Table:**

Rigaku R-AXIS RAPID diffractometer	4912 independent reflections
Radiation source: fine-focus sealed tube	4764 reflections with *I* > 2σ(*I*)
Graphite monochromator	*R*_int_ = 0.041
ω scans	θ_max_ = 27.3°, θ_min_ = 3.2°
Absorption correction: multi-scan (*ABSCOR*; Higashi, 1995)	*h* = −22→23
*T*_min_ = 0.101, *T*_max_ = 0.128	*k* = −8→8
20352 measured reflections	*l* = −23→23

### Refinement

**Table:**

Refinement on *F*^2^	Secondary atom site location: difference Fourier map
Least-squares matrix: full	Hydrogen site location: inferred from neighbouring sites
*R*[*F*^2^ > 2σ(*F*^2^)] = 0.020	H-atom parameters constrained
*wR*(*F*^2^) = 0.045	*w* = 1/[σ^2^(*F*_o_^2^) + (0.*P*)^2^ + 4.3115*P*] where *P* = (*F*_o_^2^ + 2*F*_c_^2^)/3
*S* = 1.20	(Δ/σ)_max_ = 0.002
4912 reflections	Δρ_max_ = 1.07 e Å^−^^3^
344 parameters	Δρ_min_ = −1.34 e Å^−^^3^
0 restraints	Extinction correction: *SHELXL97* (Sheldrick, 2008), Fc^*^=kFc[1+0.001xFc^2^λ^3^/sin(2θ)]^-1/4^
Primary atom site location: structure-invariant direct methods	Extinction coefficient: 0.00271 (8)

### Special details

**Table:**

Geometry. All e.s.d.'s (except the e.s.d. in the dihedral angle between two l.s. planes) are estimated using the full covariance matrix. The cell e.s.d.'s are taken into account individually in the estimation of e.s.d.'s in distances, angles and torsion angles; correlations between e.s.d.'s in cell parameters are only used when they are defined by crystal symmetry. An approximate (isotropic) treatment of cell e.s.d.'s is used for estimating e.s.d.'s involving l.s. planes.
Refinement. Refinement of *F*^2^ against ALL reflections. The weighted *R*-factor *wR* and goodness of fit *S* are based on *F*^2^, conventional *R*-factors *R* are based on *F*, with *F* set to zero for negative *F*^2^. The threshold expression of *F*^2^ > σ(*F*^2^) is used only for calculating *R*-factors(gt) *etc*. and is not relevant to the choice of reflections for refinement. *R*-factors based on *F*^2^ are statistically about twice as large as those based on *F*, and *R*- factors based on ALL data will be even larger.

### Fractional atomic coordinates and isotropic or equivalent isotropic displacement parameters (Å^2^)

**Table:**

	*x*	*y*	*z*	*U*_iso_*/*U*_eq_	
Tb1	0.373479 (7)	0.223057 (19)	0.377229 (7)	0.00937 (5)	
Tb2	0.375301 (8)	0.251927 (17)	0.896094 (8)	0.00979 (5)	
N1	0.42976 (13)	0.3322 (3)	0.27869 (13)	0.0120 (4)	
C1	0.38349 (15)	0.4121 (4)	0.21132 (16)	0.0114 (5)	
C2	0.40676 (16)	0.4340 (4)	0.14843 (16)	0.0133 (5)	
H2A	0.3734	0.4880	0.1020	0.016*	
C3	0.48211 (16)	0.3717 (4)	0.15760 (16)	0.0118 (5)	
C4	0.53247 (16)	0.3018 (4)	0.22990 (16)	0.0124 (5)	
H4A	0.5838	0.2683	0.2381	0.015*	
C5	0.50402 (16)	0.2835 (4)	0.28911 (17)	0.0117 (5)	
C6	0.30262 (16)	0.4654 (4)	0.20976 (16)	0.0129 (5)	
O1	0.25948 (12)	0.5738 (3)	0.15823 (12)	0.0172 (4)	
O2	0.28622 (12)	0.3911 (3)	0.26523 (12)	0.0199 (5)	
C7	0.50831 (16)	0.3728 (4)	0.08905 (15)	0.0118 (5)	
O3	0.45963 (13)	0.3158 (3)	0.02548 (12)	0.0209 (4)	
O4	0.57796 (12)	0.4253 (3)	0.10240 (12)	0.0161 (4)	
C8	0.55027 (16)	0.2057 (4)	0.36969 (16)	0.0128 (5)	
O5	0.62146 (12)	0.1821 (4)	0.39036 (13)	0.0265 (5)	
O6	0.50974 (12)	0.1686 (3)	0.41138 (12)	0.0177 (4)	
O7	0.36674 (12)	−0.0446 (3)	0.29048 (12)	0.0209 (5)	
H7B	0.3178	−0.0743	0.2654	0.025*	
H7A	0.3876	−0.0504	0.2574	0.025*	
O8	0.40741 (13)	0.5604 (3)	0.39763 (14)	0.0258 (5)	
H8A	0.4527	0.6096	0.4200	0.031*	
H8B	0.3682	0.5986	0.4083	0.031*	
O9	0.37823 (13)	−0.0719 (3)	0.45020 (12)	0.0213 (5)	
H9A	0.4149	−0.0984	0.4934	0.026*	
H9B	0.3420	−0.1105	0.4649	0.026*	
N2	0.38187 (14)	0.2851 (3)	0.76431 (14)	0.0118 (5)	
C9	0.31950 (16)	0.3532 (4)	0.70517 (16)	0.0127 (5)	
C10	0.31708 (17)	0.3629 (4)	0.62886 (16)	0.0148 (6)	
H10A	0.2727	0.4069	0.5885	0.018*	
C11	0.38337 (16)	0.3044 (4)	0.61552 (16)	0.0134 (5)	
C12	0.44858 (17)	0.2370 (4)	0.67751 (17)	0.0124 (6)	
H12A	0.4938	0.2006	0.6699	0.015*	
C13	0.44498 (17)	0.2250 (4)	0.75132 (17)	0.0122 (5)	
C14	0.25274 (16)	0.4193 (4)	0.72878 (16)	0.0138 (5)	
O10	0.19299 (12)	0.4879 (3)	0.67646 (12)	0.0202 (4)	
O11	0.26267 (12)	0.3988 (3)	0.79939 (12)	0.0215 (5)	
C15	0.38344 (17)	0.3049 (4)	0.53373 (16)	0.0144 (5)	
O12	0.44733 (13)	0.2845 (3)	0.52418 (13)	0.0209 (5)	
O13	0.31908 (12)	0.3159 (3)	0.47642 (12)	0.0194 (4)	
C16	0.50943 (16)	0.1457 (4)	0.82340 (16)	0.0121 (5)	
O14	0.57132 (12)	0.0860 (3)	0.81932 (12)	0.0194 (4)	
O15	0.49393 (12)	0.1463 (3)	0.88514 (11)	0.0184 (4)	
O16	0.39547 (13)	−0.0436 (3)	0.97204 (12)	0.0226 (5)	
H16B	0.4251	−0.0676	1.0162	0.027*	
H16A	0.3898	−0.1570	0.9529	0.027*	
O17	0.28287 (12)	0.3108 (4)	0.95346 (13)	0.0257 (5)	
H17B	0.2358	0.3132	0.9261	0.031*	
H17A	0.2806	0.2465	0.9929	0.031*	
O18	0.21272 (15)	0.6101 (4)	0.42001 (15)	0.0346 (6)	
H18B	0.2389	0.5106	0.4367	0.041*	
H18A	0.1688	0.5550	0.3908	0.041*	

### Atomic displacement parameters (Å^2^)

**Table:**

	*U*^11^	*U*^22^	*U*^33^	*U*^12^	*U*^13^	*U*^23^
Tb1	0.00889 (8)	0.01244 (7)	0.00678 (8)	0.00037 (4)	0.00293 (6)	0.00027 (4)
Tb2	0.00709 (8)	0.01571 (8)	0.00572 (8)	−0.00047 (4)	0.00141 (6)	0.00003 (4)
N1	0.0092 (11)	0.0155 (12)	0.0108 (11)	0.0008 (9)	0.0032 (10)	0.0015 (9)
C1	0.0086 (13)	0.0135 (13)	0.0111 (13)	0.0009 (10)	0.0023 (11)	0.0012 (10)
C2	0.0116 (13)	0.0156 (14)	0.0097 (13)	0.0002 (10)	0.0005 (11)	0.0028 (10)
C3	0.0130 (13)	0.0123 (13)	0.0105 (13)	−0.0037 (10)	0.0050 (11)	−0.0019 (9)
C4	0.0102 (13)	0.0136 (13)	0.0129 (14)	−0.0026 (10)	0.0040 (11)	−0.0009 (10)
C5	0.0099 (13)	0.0134 (13)	0.0105 (14)	−0.0017 (10)	0.0025 (12)	−0.0005 (10)
C6	0.0095 (13)	0.0166 (14)	0.0106 (13)	0.0004 (10)	0.0016 (11)	−0.0001 (10)
O1	0.0122 (10)	0.0235 (11)	0.0148 (10)	0.0044 (8)	0.0038 (9)	0.0062 (8)
O2	0.0130 (10)	0.0310 (12)	0.0174 (11)	0.0059 (8)	0.0076 (9)	0.0106 (9)
C7	0.0139 (13)	0.0121 (13)	0.0093 (13)	0.0016 (10)	0.0044 (11)	0.0018 (9)
O3	0.0208 (11)	0.0298 (12)	0.0091 (10)	−0.0040 (9)	0.0022 (9)	−0.0026 (9)
O4	0.0141 (10)	0.0208 (11)	0.0154 (10)	−0.0023 (8)	0.0080 (9)	0.0000 (8)
C8	0.0106 (13)	0.0146 (13)	0.0114 (13)	0.0002 (10)	0.0021 (12)	−0.0003 (10)
O5	0.0103 (11)	0.0439 (15)	0.0230 (13)	0.0050 (9)	0.0037 (10)	0.0105 (10)
O6	0.0130 (10)	0.0271 (12)	0.0133 (10)	0.0051 (8)	0.0051 (9)	0.0060 (8)
O7	0.0140 (10)	0.0330 (12)	0.0176 (11)	−0.0037 (9)	0.0081 (9)	−0.0103 (9)
O8	0.0238 (12)	0.0199 (12)	0.0321 (13)	−0.0043 (9)	0.0086 (11)	−0.0055 (9)
O9	0.0241 (12)	0.0237 (11)	0.0151 (11)	0.0032 (9)	0.0063 (10)	0.0067 (8)
N2	0.0100 (11)	0.0143 (11)	0.0097 (12)	−0.0011 (9)	0.0022 (10)	−0.0004 (9)
C9	0.0130 (13)	0.0137 (13)	0.0104 (13)	0.0008 (10)	0.0033 (11)	0.0008 (10)
C10	0.0136 (14)	0.0191 (14)	0.0092 (13)	0.0036 (10)	0.0013 (12)	0.0026 (10)
C11	0.0159 (14)	0.0146 (13)	0.0095 (14)	−0.0004 (11)	0.0043 (12)	−0.0023 (10)
C12	0.0125 (14)	0.0157 (14)	0.0096 (14)	0.0016 (9)	0.0049 (12)	−0.0007 (9)
C13	0.0114 (14)	0.0120 (13)	0.0128 (14)	0.0002 (10)	0.0039 (12)	−0.0005 (10)
C14	0.0130 (13)	0.0150 (13)	0.0127 (13)	0.0021 (10)	0.0038 (12)	−0.0001 (10)
O10	0.0159 (10)	0.0283 (12)	0.0122 (10)	0.0099 (9)	0.0006 (9)	0.0038 (8)
O11	0.0154 (11)	0.0391 (13)	0.0102 (10)	0.0086 (9)	0.0052 (9)	0.0039 (9)
C15	0.0191 (15)	0.0151 (14)	0.0095 (13)	0.0016 (11)	0.0060 (12)	−0.0002 (10)
O12	0.0166 (11)	0.0344 (12)	0.0119 (11)	0.0012 (9)	0.0056 (10)	−0.0019 (9)
O13	0.0169 (11)	0.0307 (12)	0.0095 (10)	0.0043 (9)	0.0036 (9)	−0.0018 (9)
C16	0.0107 (13)	0.0140 (13)	0.0099 (13)	−0.0010 (10)	0.0021 (11)	−0.0005 (10)
O14	0.0131 (10)	0.0293 (12)	0.0167 (11)	0.0063 (8)	0.0067 (9)	0.0022 (8)
O15	0.0125 (10)	0.0332 (12)	0.0091 (10)	0.0071 (8)	0.0035 (9)	0.0043 (8)
O16	0.0265 (12)	0.0206 (11)	0.0132 (11)	0.0038 (9)	−0.0011 (10)	0.0027 (8)
O17	0.0107 (10)	0.0488 (15)	0.0182 (12)	0.0049 (10)	0.0060 (10)	0.0052 (10)
O18	0.0298 (14)	0.0369 (15)	0.0294 (14)	0.0125 (11)	0.0023 (12)	−0.0028 (11)

### Geometric parameters (Å, º)

**Table:**

Tb1—O1^i^	2.508 (2)	O4—Tb2^iii^	2.387 (2)
Tb1—O2	2.400 (2)	C8—O5	1.232 (3)
Tb1—O6	2.378 (2)	C8—O6	1.287 (3)
Tb1—O7	2.426 (2)	O7—H7B	0.8706
Tb1—O8	2.406 (2)	O7—H7A	0.8392
Tb1—O9	2.431 (2)	O8—H8A	0.8514
Tb1—O12	2.590 (2)	O8—H8B	0.8583
Tb1—O13	2.489 (2)	O9—H9A	0.8538
Tb1—N1	2.534 (2)	O9—H9B	0.8526
Tb2—O3^ii^	2.365 (2)	N2—C13	1.339 (4)
Tb2—O4^iii^	2.387 (2)	N2—C9	1.343 (4)
Tb2—O10^iv^	2.336 (2)	C9—C10	1.404 (4)
Tb2—O11	2.403 (2)	C9—C14	1.523 (4)
Tb2—O15	2.384 (2)	C10—C11	1.394 (4)
Tb2—O16	2.430 (2)	C10—H10A	0.9300
Tb2—O17	2.358 (2)	C11—C12	1.397 (4)
Tb2—N2	2.507 (2)	C11—C15	1.520 (4)
N1—C1	1.342 (3)	C12—C13	1.400 (4)
N1—C5	1.351 (4)	C12—H12A	0.9300
C1—C2	1.396 (4)	C13—C16	1.521 (4)
C1—C6	1.525 (4)	C14—O10	1.257 (3)
C2—C3	1.402 (4)	C14—O11	1.263 (3)
C2—H2A	0.9300	O10—Tb2^vii^	2.336 (2)
C3—C4	1.402 (4)	C15—O12	1.262 (4)
C3—C7	1.522 (4)	C15—O13	1.265 (4)
C4—C5	1.390 (4)	C16—O14	1.241 (3)
C4—H4A	0.9300	C16—O15	1.282 (3)
C5—C8	1.517 (4)	O16—H16B	0.8149
C6—O1	1.241 (3)	O16—H16A	0.8500
C6—O2	1.285 (3)	O17—H17B	0.8253
O1—Tb1^v^	2.508 (2)	O17—H17A	0.8714
C7—O3	1.250 (3)	O18—H18B	0.8298
C7—O4	1.266 (3)	O18—H18A	0.8749
O3—Tb2^vi^	2.365 (2)		
			
O6—Tb1—O2	127.28 (7)	C2—C1—C6	124.2 (2)
O6—Tb1—O8	85.70 (8)	C1—C2—C3	117.8 (2)
O2—Tb1—O8	73.64 (8)	C1—C2—H2A	121.1
O6—Tb1—O7	80.97 (7)	C3—C2—H2A	121.1
O2—Tb1—O7	86.64 (8)	C2—C3—C4	119.5 (2)
O8—Tb1—O7	142.08 (8)	C2—C3—C7	120.4 (2)
O6—Tb1—O9	84.53 (7)	C4—C3—C7	120.0 (2)
O2—Tb1—O9	139.62 (7)	C5—C4—C3	118.6 (3)
O8—Tb1—O9	140.44 (8)	C5—C4—H4A	120.7
O7—Tb1—O9	73.38 (8)	C3—C4—H4A	120.7
O6—Tb1—O13	121.38 (7)	N1—C5—C4	121.7 (3)
O2—Tb1—O13	101.12 (7)	N1—C5—C8	113.2 (2)
O8—Tb1—O13	77.68 (8)	C4—C5—C8	125.1 (3)
O7—Tb1—O13	138.92 (7)	O1—C6—O2	125.8 (3)
O9—Tb1—O13	75.12 (7)	O1—C6—C1	120.0 (2)
O6—Tb1—O1^i^	146.61 (7)	O2—C6—C1	114.2 (2)
O2—Tb1—O1^i^	72.52 (7)	C6—O1—Tb1^v^	137.01 (18)
O8—Tb1—O1^i^	127.62 (7)	C6—O2—Tb1	127.35 (17)
O7—Tb1—O1^i^	73.14 (7)	O3—C7—O4	126.3 (3)
O9—Tb1—O1^i^	68.26 (8)	O3—C7—C3	116.7 (2)
O13—Tb1—O1^i^	71.10 (7)	O4—C7—C3	117.0 (2)
O6—Tb1—N1	64.09 (7)	C7—O3—Tb2^vi^	170.3 (2)
O2—Tb1—N1	63.37 (7)	C7—O4—Tb2^iii^	127.23 (17)
O8—Tb1—N1	70.91 (8)	O5—C8—O6	125.2 (3)
O7—Tb1—N1	71.31 (7)	O5—C8—C5	119.6 (3)
O9—Tb1—N1	135.54 (7)	O6—C8—C5	115.2 (2)
O13—Tb1—N1	147.75 (8)	C8—O6—Tb1	126.89 (18)
O1^i^—Tb1—N1	123.80 (7)	Tb1—O7—H7B	108.7
O6—Tb1—O12	70.06 (7)	Tb1—O7—H7A	126.8
O2—Tb1—O12	138.79 (8)	H7B—O7—H7A	105.2
O8—Tb1—O12	70.86 (8)	Tb1—O8—H8A	127.8
O7—Tb1—O12	134.56 (7)	Tb1—O8—H8B	98.2
O9—Tb1—O12	69.76 (7)	H8A—O8—H8B	121.3
O13—Tb1—O12	51.34 (7)	Tb1—O9—H9A	123.7
O1^i^—Tb1—O12	114.86 (7)	Tb1—O9—H9B	124.9
N1—Tb1—O12	121.18 (7)	H9A—O9—H9B	94.1
O6—Tb1—C15	95.68 (8)	C13—N2—C9	119.7 (2)
O2—Tb1—C15	123.08 (8)	C13—N2—Tb2	120.68 (19)
O8—Tb1—C15	74.82 (8)	C9—N2—Tb2	119.46 (18)
O7—Tb1—C15	141.56 (8)	N2—C9—C10	122.5 (2)
O9—Tb1—C15	68.18 (8)	N2—C9—C14	113.9 (2)
O13—Tb1—C15	25.73 (8)	C10—C9—C14	123.6 (2)
O1^i^—Tb1—C15	91.86 (8)	C11—C10—C9	117.8 (3)
N1—Tb1—C15	141.02 (8)	C11—C10—H10A	121.1
O12—Tb1—C15	25.80 (8)	C9—C10—H10A	121.1
O10^iv^—Tb2—O17	94.09 (8)	C10—C11—C12	119.4 (2)
O10^iv^—Tb2—O3^ii^	137.68 (8)	C10—C11—C15	120.4 (2)
O17—Tb2—O3^ii^	79.58 (8)	C12—C11—C15	120.2 (2)
O10^iv^—Tb2—O15	91.41 (8)	C11—C12—C13	119.1 (3)
O17—Tb2—O15	158.69 (7)	C11—C12—H12A	120.5
O3^ii^—Tb2—O15	82.50 (8)	C13—C12—H12A	120.5
O10^iv^—Tb2—O4^iii^	148.23 (7)	N2—C13—C12	121.4 (3)
O17—Tb2—O4^iii^	98.76 (8)	N2—C13—C16	113.4 (2)
O3^ii^—Tb2—O4^iii^	73.56 (7)	C12—C13—C16	125.2 (2)
O15—Tb2—O4^iii^	87.06 (7)	O10—C14—O11	126.2 (3)
O10^iv^—Tb2—O11	76.71 (8)	O10—C14—C9	117.3 (2)
O17—Tb2—O11	72.40 (7)	O11—C14—C9	116.6 (2)
O3^ii^—Tb2—O11	137.43 (8)	C14—O10—Tb2^vii^	150.1 (2)
O15—Tb2—O11	128.91 (7)	C14—O11—Tb2	124.96 (18)
O4^iii^—Tb2—O11	79.69 (8)	O12—C15—O13	121.2 (3)
O10^iv^—Tb2—O16	67.14 (8)	O12—C15—C11	119.3 (3)
O17—Tb2—O16	82.04 (8)	O13—C15—C11	119.4 (2)
O3^ii^—Tb2—O16	70.54 (8)	O12—C15—Tb1	63.24 (15)
O15—Tb2—O16	81.17 (8)	O13—C15—Tb1	58.67 (14)
O4^iii^—Tb2—O16	143.32 (7)	C11—C15—Tb1	168.1 (2)
O11—Tb2—O16	133.68 (8)	C15—O12—Tb1	90.96 (18)
O10^iv^—Tb2—N2	73.74 (7)	C15—O13—Tb1	95.60 (16)
O17—Tb2—N2	137.12 (8)	O14—C16—O15	125.0 (3)
O3^ii^—Tb2—N2	136.26 (8)	O14—C16—C13	119.9 (2)
O15—Tb2—N2	64.13 (8)	O15—C16—C13	115.2 (2)
O4^iii^—Tb2—N2	77.11 (7)	C16—O15—Tb2	126.57 (18)
O11—Tb2—N2	64.84 (7)	Tb2—O16—H16B	130.9
O16—Tb2—N2	126.24 (8)	Tb2—O16—H16A	124.3
C1—N1—C5	119.5 (2)	H16B—O16—H16A	99.5
C1—N1—Tb1	120.52 (17)	Tb2—O17—H17B	119.8
C5—N1—Tb1	119.27 (18)	Tb2—O17—H17A	124.3
N1—C1—C2	122.6 (2)	H17B—O17—H17A	99.1
N1—C1—C6	113.1 (2)	H18B—O18—H18A	98.3

Symmetry codes: (i) −*x*+1/2, *y*−1/2, −*z*+1/2; (ii) *x*, *y*, *z*+1; (iii) −*x*+1, −*y*+1, −*z*+1; (iv) −*x*+1/2, *y*−1/2, −*z*+3/2; (v) −*x*+1/2, *y*+1/2, −*z*+1/2; (vi) *x*, *y*, *z*−1; (vii) −*x*+1/2, *y*+1/2, −*z*+3/2.

### Hydrogen-bond geometry (Å, º)

**Table:**

*D*—H···*A*	*D*—H	H···*A*	*D*···*A*	*D*—H···*A*
O7—H7*A*···O14^viii^	0.84	1.86	2.700 (3)	174.4
O7—H7*B*···O2^i^	0.87	1.80	2.651 (3)	164.8
O8—H8*A*···O12^iii^	0.85	1.89	2.741 (3)	176.0
O8—H8*B*···O9^ix^	0.86	2.39	2.844 (3)	113.0
O9—H9*A*···O5^viii^	0.85	2.56	3.057 (3)	119.0
O9—H9*A*···O6^viii^	0.85	1.86	2.711 (3)	176.0
O9—H9*B*···O5^viii^	0.85	2.56	3.057 (3)	118.0
O16—H16*A*···O4^viii^	0.85	2.30	3.101 (3)	156.2
O16—H16*B*···O15^x^	0.81	1.96	2.766 (3)	171.0
O17—H17*A*···O18^iv^	0.87	1.84	2.705 (4)	173.9
O17—H17*B*···O5^xi^	0.82	1.95	2.759 (3)	163.6
O18—H18*A*···O14^xii^	0.87	2.04	2.911 (4)	175.7
O18—H18*B*···O13	0.83	1.92	2.745 (4)	167.1

Symmetry codes: (i) −*x*+1/2, *y*−1/2, −*z*+1/2; (iii) −*x*+1, −*y*+1, −*z*+1; (iv) −*x*+1/2, *y*−1/2, −*z*+3/2; (viii) −*x*+1, −*y*, −*z*+1; (ix) *x*, *y*+1, *z*; (x) −*x*+1, −*y*, −*z*+2; (xi) *x*−1/2, −*y*+1/2, *z*+1/2; (xii) *x*−1/2, −*y*+1/2, *z*−1/2.
